# Assessment of traditional and non-traditional risk factors for premature atherosclerosis in children with juvenile dermatomyositis and pediatric controls

**DOI:** 10.1186/s12969-020-0415-5

**Published:** 2020-03-18

**Authors:** Dawn M. Wahezi, Emily J. Liebling, Jaeun Choi, Marija Dionizovik-Dimanovski, Qi Gao, Jillian Parekh

**Affiliations:** 1 Children’s Hospital at Montefiore, Bronx, NY USA; 2 Albert Einstein College of Medicine, Bronx, NY USA

**Keywords:** Pediatric dermatomyositis, Cardiovascular disease, Premature atherosclerosis

## Abstract

**Background:**

Children with juvenile dermatomyositis (JDM), the most common inflammatory myopathy of childhood, may be at increased risk of premature atherosclerosis given a host of traditional and non-traditional risk factors. The primary aim of this study was to determine the underlying frequency of premature atherosclerosis in children with JDM compared to pediatric controls using flow-mediated dilation as a measure of endothelial function.

**Methods:**

Children and adolescents with and without JDM were evaluated for traditional atherosclerotic risk factors and assessment of endothelial function, using Endothelial Pulse Amplitude Testing (Endo-PAT).

**Results:**

In this study, 75% of pediatric controls were of Black or Hispanic descent (compared to 55% in the JDM group) and 70% were found to live in a household with a medium income less than $50,000/year (compared to 45% in the JDM group). Among traditional atherogenic risk factors, lipoprotein A appeared to be different between controls and JDM patients (66 nmol/L and 16.5 nmol/L, respectively). Using a reactive hyperemia index (RHI) < 1.67 as evidence of endothelial dysfunction, 75% of controls were defined as having endothelial dysfunction compared to 50% in JDM group. When controlled for lipoprotein A as an atherogenic confounder, JDM patients were found to have a 41% increase in RHI, thus indicating less endothelial dysfunction compared to controls.

**Conclusions:**

In this study, we have shown that atherogenic risk factors are present in the pediatric population and may be associated with endothelial dysfunction, even at very young ages. Despite increasing concerns that children with rheumatologic disorders may be at increased risk of developing premature atherosclerosis, traditional and sociodemographic features may play a greater role in the ultimate development of cardiovascular disease.

## Background

Cardiovascular disease (CVD) is a leading cause of morbidity and mortality in the adult population. There is increasing evidence to suggest that atherosclerosis begins in childhood, progresses silently for many years and only becomes apparent once clinical manifestations, including myocardial infarction and/or cerebrovascular accident, occur [1, 2]. Children with rheumatologic disorders may be at increased risk of developing premature atherosclerosis due to both disease specific risk factors, such as chronic inflammation, corticosteroid burden, elevated homocysteine levels and underlying vasculopathy; as well as a host of traditional risk factors, including dyslipidemia, insulin resistance, obesity and hypertension [3–7]. Premature atherosclerosis has been demonstrated among young patients with systemic lupus erythematosus (SLE) and Kawasaki disease (KD), using non-invasive ultrasound techniques including carotid intima-media thickness (CIMT) and flow-mediated dilation (FMD) [4, 8]. This finding is not surprising given the evidence that atherosclerosis is, in large part, an inflammatory disorder initiated and propagated by both humoral and cellular immunity [9, 10].

Cardiovascular manifestations of juvenile dermatomyositis (JDM), the most common idiopathic inflammatory myopathy of childhood, include acquired structural abnormalities, ventricular dysfunction, arrhythmias, myocarditis, and hypertension [11–17], with myocardial infarction acting as a rare cause of death [11, 13, 18, 19]. JDM shares the same atherogenic risk factors and vasculopathic features as those of other rheumatologic illnesses, with the added component of lipodystrophy and its related metabolic derangements [15, 20, 21]. Moreover, higher rates of traditional atherosclerotic risk factors have been reported in adult patients with idiopathic inflammatory myopathies as compared to the general population [12, 17, 22].

A potential consequence of the systemic vasculopathy in JDM is endothelial dysfunction, a broad term denoting impaired vasodilation secondary to diminished production or availability of nitric oxide (NO) [9]. Recognized as an early physiologic precursor of atherosclerosis, endothelial dysfunction can long precede vessel structure changes, which allows it to serve as an independent predictor of future CVD [9, 23]. Prior investigations of endothelial dysfunction in children have used non-invasive ultrasound techniques, such as brachial artery FMD and pulse wave velocity (PWV) as validated surrogate outcome markers of early atherosclerosis [20, 24]. These methods correlate with cardiovascular events and severity of coronary artery disease in adults, as confirmed by angiography [25–29]. Further substantiating these tools in children is the association between reduced FMD and the presence of atherogenic risk factors in pediatric patients with KD, familial hypercholesterolemia, type I diabetes mellitus (T1DM), and obesity [8, 30–36]. Despite significant advances in ultrasonographic technology, limitations surround its use, particularly in children [37]. Endothelial Pulse Amplitude Testing (Endo-PAT) is an FDA approved method of detecting endothelial dysfunction, that is inexpensive, non-invasive, reproducible, and operator independent. It assesses post-occlusive vasodilatory response in the digital arteries and serves as an accurate measure of endothelial function in both central and peripheral circulation in adults [38, 39].

There is a paucity of data regarding cardiovascular outcomes in children with JDM, and to date, premature atherosclerosis has not been evaluated in these patients. Identification of the earliest stages of the atherosclerotic process in patients with chronic inflammation is critical to the prevention of the long-term morbidity and mortality associated with CVD. The primary aim of this study was to determine the underlying frequency of premature atherosclerosis and atherogenic risk factors in children with JDM, as compared to pediatric controls, using flow-mediated dilation as a measure of endothelial function. The secondary aim was to evaluate the potential association of endothelial dysfunction with these atherogenic risk factors.

## Methods

### Study participants and recruitment

Children, adolescents, and young adults, age 2–22 years, with a diagnosis of probable or definite JDM, as determined by Bohan and Peter criteria, seen in the pediatric rheumatology clinic at the Children’s Hospital at Montefiore (CHAM) from September 2014 to September 2016, were selected for inclusion in the study [40]. Retrospective clinical and laboratory information were obtained via chart review to determine disease specific characteristics including date of diagnosis, muscle enzymes, disease duration, medication usage, and disease manifestations (including calcinosis, lipodystrophy, skin ulcers, cardiopulmonary or gastrointestinal involvement). Pediatric controls included patients with no known co-existing chronic medical conditions, seen in the general pediatric clinic at the Family Care Center, Montefiore Medical Center. Controls were manually matched based on comparison to patients with JDM with regards to age, gender, race and body mass index (BMI), in an attempt to balance the groups. Exclusion criteria included (1) a diagnosis of chronic illness other than JDM (2) known CVD (3) current use of medication that alters lipid metabolism or endothelial function and (4) current smoking. Twenty matched pairs were recruited. Annual medium household income by zip-code was calculated for all participants using U.S. Census Bureau, 2012–2016 American Community Survey. Values were categorized in increments of $25,000 to allow for comparison. The study protocol and written informed consent procedures were approved by the Institutional Review Board of the Einstein Human Research Protection Program of the Albert Einstein College of Medicine – Montefiore Medical Center (Reference # 11–10-376E).

### Atherosclerotic risk factor assessment

Anthropometric assessments of all subjects were performed including weight, height, BMI, pulse rate, and blood pressure. Smoking history, personal and familial history of CVD, hypertension, and diabetes mellitus were obtained by demographic questionnaire. After an overnight fast, blood specimens were collected for the following evaluations: total cholesterol (TC), low-density lipoprotein (LDL), high-density lipoprotein (HDL), triglycerides (TG), insulin, glucose, hemoglobin A1C, high-sensitivity C-reactive protein (hsCRP), lipoprotein A, homocysteine, apolipoprotein A1, and apolipoprotein B. Assessment of insulin resistance was performed using the Homeostatic Model Assessment for Insulin Resistance (HOMA-IR) with the following formula: *HOMA-IR = fasting insulin x fasting glucose / 22.5* [41].

### JDM disease activity and severity assessments

Additional disease specific laboratory evaluations were performed on subjects with JDM including creatine phosphokinase (CPK), lactate dehydrogenase (LDH), aspartate aminotransferase (AST), aldolase, and von-Willebrand factor (vWF) antigen. Values were determined to be normal based on local lab cutoffs. Validated disease activity measures for JDM were performed, including the Childhood Myositis Assessment Scale (CMAS, range 0–52) and Disease Activity Score (DAS, range 0–20), the latter assessing both muscle and skin components [42, 43]. Long-term damage was assessed using the International Myositis Assessment and Clinical Studies Group (IMACS) Myositis Damage Index (MDI), evaluated in this study using the extent of damage scoring system (potential range 0–35 in children age < 12 years, 0–37 in adolescents and 0–38 in adults) [44, 45].

### Flow mediated dilation

*Endo-PAT.* Assessment of endothelial function was performed in all patients using Endo-PAT (Itamar Medical Ltd., Caesarea, Israel), which combines features of traditional FMD with beat-to-beat plethysmographic recordings of digital arterial pulse wave amplitude using pneumatic probes. Subjects were required to avoid exercise, caffeine, and foods with high fat content one day prior to testing, and to engage in an eight hour overnight fast. Fingernails were trimmed to improve accuracy of the study. Prior to the onset of the study, patients rested comfortably on an examination bed in a warm room for 15 min to allow for a relaxed cardiovascular steady-state. Baseline pulse wave amplitude measurements at the distal tip of both index fingers were recorded over five minutes. To measure reactive hyperemia, the brachial artery of the right arm was occluded with a blood pressure cuff inflated to 40–60 mmHg above systolic pressure for five minutes with continued recording. This was followed by rapid cuff deflation and subsequent five-minute recording time. The contralateral arm served as the control for the entire pulse wave amplitude recording period. The Endo-PAT / reactive hyperemia index (RHI) was calculated using proprietary software: it is the ratio of the average post-occlusion pulse amplitude to the average pre-occlusion pulse amplitude baseline, multiplied by a baseline correction factor. Prior evaluation of discomfort associated with this procedure resulted in a median pain score of one on the Wong-Baker Faces Pain Scale [46].

Reduced values of RHI indicate a reduced reactive hyperemic response and thus increased likelihood of endothelial dysfunction. Values of RHI < 1.67 were defined as evidence of endothelial dysfunction, as determined by Bonetti et al., to yield 82% sensitivity and 77% specificity in the identification of adult patients with coronary endothelial dysfunction [38]. Trials of Endo-PAT in children yielded a mean RHI of 1.78 in healthy subjects, as compared to 1.50 in the severely obese and 1.63 in T1DM, but no discrete parameters have yet been identified in the pediatric population [32, 46].

### Data analysis

All data analyses were preceded by extensive checking and verification to identify and resolve reasons for missing data, inconsistencies, and out-of-range values. Descriptive analyses were performed on the demographic and clinical characteristics of study participants, variables related to JDM status (case/control) and endothelial function. Bivariate associations of categorical variables with JDM status were evaluated by the Chi-square or Fisher’s exact test; and mean and median levels of continuous variables were estimated and compared between JDM patients and pediatric controls using the two-sample T-test or Wilcoxon rank sum test, as dictated by distribution of the data. Linear regression models were used to compare JDM patients and pediatric controls with respect to endothelial function measure (i.e. RHI). The outcome variable, RHI, was log-transformed due to non-normally distributed data and the results are reported as a ratio of RHI (i.e. percent change in RHI) for ease of interpretation. There was an issue of limited sample size which did not support a multivariable analysis adjusting for multiple risk factors together, and therefore exploratory analyses were performed through models individually constructed for each atherogenic risk factor uncorrected to assess for an interaction with JDM status on the outcome. Based on the results from the exploratory uncorrected tests, additional exploratory stratification by JDM status was conducted for the individual risk factors. Additional subgroup analysis of JDM patients was performed to evaluate the usage of medications on RHI by Wilcoxon rank sum tests due to limited sample sizes of medication groups and non-normality of the RHI data. *P*-value less than 0.05 was considered statistically significant. Data were analyzed by using SAS software (version 9.4; SAS Institute, Inc., Cary, NC).

## Results

### Clinical characteristics of study participants

**Fig. 1:**
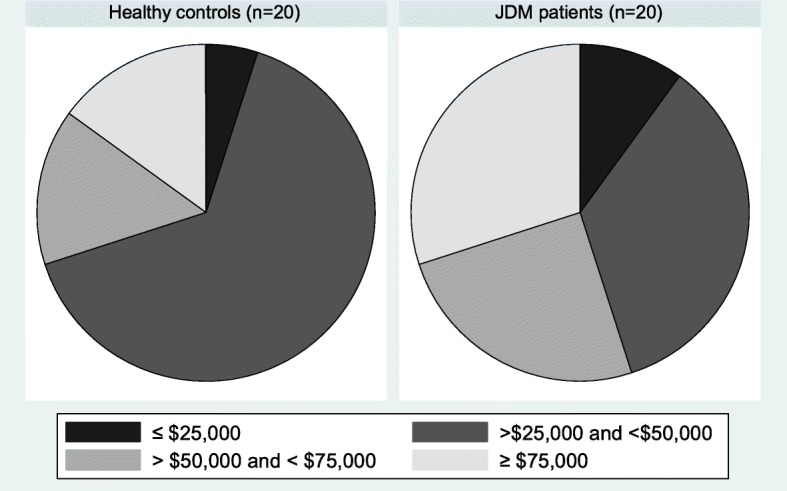
Annual medium household income category in JDM patients versus pediatric controls

Table 1 lists the demographic and clinical features of the study participants at the time of evaluation in this study. Overall, the mean age was 12.4 ± 4.1 years (range 6–22) with a female preponderance (70%). Race and ethnicity were self-reported with 35% of total participants identifying as White, non-Hispanic, 20% as Black, non-Hispanic and 45% as Hispanic. There were no statistically significant differences between groups with regards to age, gender, race or BMI; however, 55% of pediatric controls reported Hispanic ethnicity compared to 35% in the JDM group. Annual medium household income by zip-code was reviewed for each study participant; 70% of pediatric controls and 45% of JDM patients were found to live in a household with a medium income less than $50,000/year (Fig. 1).

**Table 1:** Clinical and laboratory data in JDM patients and pediatric controls (*n* = 40)^a^

	Total (*n* = 40)	Pediatric controls (*n* = 20)	JDM patients (*n* = 20)	*p*-value
Age (years)	12.4 ± 4.1	12.7 ± 3.9	12.1 ± 4.4	0.651
Female gender	28 (70%)	14 (70%)	14 (70%)	> 0.999
Race				0.392
White, non-Hispanic	14 (35%)	5 (25%)	9 (45%)	
Black, non-Hispanic	8 (20%)	4 (20%)	4 (20%)	
Hispanic	18 (45%)	11 (55%)	7 (35%)	
BMI	20.9 ± 5	20.9 ± 4.9	21 ± 5.1	0.950
BMI category				> 0.999
Healthy	24 (60%)	12 (60%)	12 (60%)	
Overweight/Obese	16 (40%)	8 (40%)	8 (40%)	
Positive cardiac family history	23 (58%)	12 (60%)	11 (58%)	0.894
Systolic blood pressure (mmHg)	109.8 ± 10.8	106.9 ± 10.5	112.8 ± 10.5	0.081
Diastolic blood pressure (mmHg)	66 ± 7	63.7 ± 6.9	68.4 ± 6.6	**0.035**
Total cholesterol (mg/dL)	159.1 ± 32.9	163.1 ± 29.1	154.8 ± 36.7	0.441
LDL (mg/dL)	87.6 ± 25.5	92.8 ± 23.5	82.3 ± 27.0	0.203
HOMA-IR^b^	2.1 [1.4, 3.1]	2 [1.7, 2.8]	2.1 [1.2, 3.3]	0.922
Hemoglobin A1c	5.5 ± 0.3	5.6 ± 0.3	5.4 ± 0.3	0.084
Lipoprotein A (nmol/L)^c^	46 [14,87]	66 [24,91]	16.5 [10, 70]	0.055
Apolipoprotein B/A1 ratio	0.5 ± 0.1	0.53 ± 0.1	0.46 ± 0.1	0.082
hsCRP (mg/L)	0.2 [0.1, 0.7]	0.3 [0.2, 0.8]	0.2 [0.1, 0.4]	0.178
RHI	1.57 [1.2,1.9]	1.43 [1.2, 1.7]	1.72 [1.3, 2.4]	0.148
Abnormal RHI < 1.67	25 (63%)	15 (75%)	10 (50%)	0.103
Log RHI	0.45 ± 0.33	0.36 ± 0.24	0.54 ± 0.39	0.089

^a^ Continuous variables expressed as mean ± standard deviation or median [interquartile range]. Categorical variables expressed as frequency (percentages)

^b^ Homeostatic Model Assessment for Insulin Resistance (HOMA-IR): *HOMA-IR = fasting insulin x fasting glucose / 22.5* [43]

^c^ Only evaluated in 31 participants (17 pediatric controls and 14 JDM patients)

JDM patients had a median disease duration of 44 months [IQR: 27, 75] with minimal evidence of active disease and damage (median CMAS = 52, range 47–52; median DAS 0, range 0–5; median MDI (extent) =0, range 0–3). CPK, LDH, AST and aldolase were normal in over 90% of JDM patients (Table 2). Among the JDM patients, at the time of the study, 7 were currently on prednisone (35%) (with doses ranging from 0.1–0.7 mg/kg/day), 13 on hydroxychloroquine (65%), 15 on methotrexate (75%) and 9 on IVIG (45%). None of the JDM patients had previously known cardiovascular disease.

**Table 2:** Disease activity assessment in JDM patients (*n* = 20)

Measure of Disease Activity	Median [IQR]	Patients with normal values*
CPK (U/L)	95 [71, 153]	19 (95%)
LDH (U/L)	213 [176, 243]	19 (95%)
AST (U/L)	24 [22, 27]	18 (90%)
Aldolase (U/L)	6.4 [5.6, 7.3]	19 (95%)
VWF antigen (%)	119 [72, 149]	12 (63%)
CMAS	52 [50, 52]	13 (65%)
DAS	0 [0, 2]	11 (55%)

### Bivariate analyses of endothelial function and atherogenic risk factors between JDM cases and pediatric controls

Among the total study population of pediatric controls and JDM patients, 63% of participants had endothelial dysfunction (as defined by adult Endo-PAT cutoff < 1.67), with 75% in pediatric controls and 50% in JDM patients (Table 1). Pediatric controls had lower median RHI than JDM patients (1.43 [1.2, 1.7] versus 1.72 [1.3, 2.4] respectively) and higher endothelial dysfunction, however, these results were not significantly different between groups. In evaluation of atherogenic risk factors, patients with JDM had significantly higher diastolic blood pressure compared to pediatric controls (*p* = 0.035). Among traditional atherogenic risk factors, lipoprotein A appeared to be different with higher levels in pediatric controls than JDM patients (66 nmol/L versus 16.5 nmol/L respectively; *p* = 0.055) (Table 1).

### Exploratory univariate assessment of endothelial function in JDM

The association of JDM status with endothelial function was evaluated using individual linear models controlling for potentially confounding atherogenic risk factors. JDM status appeared to be a significant predictor for increased RHI when controlled for lipoprotein A (Table 3, *p* = 0.006) or hsCRP (Table 4, *p* = 0.048). More specifically, when controlled for lipoprotein A as an atherogenic confounder, JDM patients were found to have a 41% increase in reactive hyperemia index (RHI), thus indicating less endothelial dysfunction compared to pediatric controls. In addition, we conducted the model adjusting for both lipoprotein A and hsCRP, however, the Likelihood Ratio Test showed that the model controlling for lipoprotein A only is more appropriate than the bigger model controlling for both, which implies that lipoprotein A is the dominating confounder and that the role of hsCRP as a confounder is more pronounced without lipoprotein A. Therefore, the results from the individual models controlling for lipoprotein A or hsCRP are presented separately.

**Table 3:** Association between JDM status and endothelial function when controlling for lipoprotein A (*n* = 31)

	Ratio of RHI	95% CI	*p*-value
JDM status	1.41	1.12, 1.79	**0.006**
Lipoprotein A	1.00	0.99, 1.00	0.218

**Table 4:** Association between JDM status and endothelial function when controlling for hsCRP (*n* = 39)

	Ratio of RHI	95% CI	*p*-value
JDM status	1.24	1.00, 1.52	**0.048**
hsCRP	1.00	0.97, 1.04	0.996

### Association between endothelial function and BMI

The association of BMI and RHI was investigated by JDM status-stratified analyses due to a statistically significant interaction with BMI and JDM status on RHI (*p* < 0.0001). After stratification by JDM status, BMI was found to be a significant predictor of increased RHI in JDM patients (*p* < 0.0001), but not in pediatric controls (*p* = 0.334) (Table 5).

**Table 5:** Association between BMI and RHI in patients stratified by JDM status

	JDM patients (***n*** = 20)			Pediatric controls (***n*** = 20)		
	Ratio of RHI	95% CI	*p*-value	Ratio of RHI	95% CI	*p*-value
BMI	1.06	1.04, 1.09	**< 0.0001**	0.99	0.97,1.01	0.334

### Evaluation of endothelial function within JDM participants

The majority of JDM patients showed minimal evidence of disease activity, as demonstrated by greater than 90% with normal muscle enzymes, median CMAS = 52 and median DAS = 0. Eight patients were found to have both a CMAS = 52 and DAS = 0. Among these patients, 3 (37.5%) were found to have an abnormal RHI < 1.67 compared to 7 (58.3%) of the JDM patients with either an abnormal CMAS or DAS, however these results were not statistically significant (*p* = 0.650). There were no statistically significant differences in RHI with regards to MDI or when comparing current medication usage with those off the respective medication including prednisone, hydroxychloroquine, methotrexate, IVIG.

## Discussion

Children with JDM, similar to other rheumatic diseases, may be at increased risk of premature atherosclerosis due to a host of traditional and non-traditional atherogenic risk factors. Typical contributing factors to poor outcomes in JDM include younger age at presentation, high initial serum CPK, longer disease duration, and complications including calcinosis and lipodystrophy [47]. It is unknown, however, to what extent these contribute to cardiovascular prognosis. To our knowledge, this is the first study to evaluate the frequency of premature atherosclerosis, as measured by endothelial dysfunction, in a racially diverse pediatric population with JDM.

Although there are no prior studies examining the prevalence of premature atherosclerosis in children with JDM, two small case-control studies have been performed assessing the cardiovascular outcomes of patients with history of JDM in childhood. The first involved 59 patients with JDM (mean age 16.8, range 2–38 years), in which a higher prevalence of hypertension and ventricular diastolic dysfunction was seen as compared to healthy age- and gender- matched controls [16]. In this study, a significant association was found between cardiac dysfunction and early disease activity (one-year post diagnosis). A second pilot study found increased cardiovascular risk factors and evidence suggestive of premature atherosclerosis in 8 *adults* with a prior history of JDM (median age: 38 years old), as compared to healthy controls. In this study, the adult patients with a history of JDM exhibited increased atherogenic risk factors (including higher blood pressure, lower adiponectin, less upper arm fat and increased proinflammatory oxidized HDL), as well as significantly increased CIMT and decreased FMD, as compared to healthy controls [48].

In our study, we found a statistically significant difference in endothelial function between JDM patients and pediatric controls when controlled for hsCRP or lipoprotein A, whereas our population of pediatric controls appeared to demonstrate a reduced post-occlusive hyperemic response (i.e. reduced RHI), and thus worse endothelial function, as compared to JDM patients. Although of borderline statistical significance, pediatric controls appeared to have higher levels of lipoprotein A, one of traditional atherogenic risk factors, which was an important confounder in our results. Lipoprotein A, independent of other lipids, has been associated with abnormal FMD and is an independent risk factor for premature atherosclerosis by causing impaired endothelium-dependent vasodilation of angiographically normal coronary arteries [31, 49, 50]. Similarly, hsCRP which was slightly higher in control patients was a confounder in the association between JDM status and endothelial dysfunction. This raises that question of whether the true inflammatory burden in JDM may be less than that of other rheumatic diseases (such as SLE) or whether achieving adequate disease control (as was seen in our patient sample), impacts overall endothelial function.

Interestingly, BMI was positively associated with RHI only in the JDM cohort, implying better endothelial function in the overweight/obese patients. This finding could possibly be explained by the potential beneficial effects that corticosteroids may have had on the vascular endothelium in JDM patients, while simultaneously contributing to their increasing BMI. A recent study highlights the conflicting impact that corticosteroids may have on endothelial function in patients with inflammatory diseases; whereas direct deleterious effects on the endothelium may be seen, these may be outweighed by the positive impact of reducing vascular inflammation [51]. We speculate that in the JDM patients, BMI was likely elevated due to a known side effect of steroid toxicity. Therefore, JDM patients with increasing BMI may have been simultaneously benefiting from reduced vascular inflammation and potentially reduced endothelial dysfunction. This theory may be supported by the fact that the majority of our JDM patients demonstrated minimal evidence of disease activity at the time of the study. In contrast, in control subjects, BMI was not found to be protective and may represent a group of patients with dietary/familial induced obesity and thus potentially increased traditional atherosclerotic risk factors.

An additional consideration in the interpretation of our results is the impact that race, income and social disparities have on chronic disease and future comorbidities. The participants in this study primarily represent a population of Black and Hispanic youth from low income households in an inner-city clinic. Previous studies have demonstrated that racial and ethnic disparities are associated with worse morbidity and adult outcomes in children with chronic disease, including JDM [52–54]. Although it is difficult to apply adult cutoffs to children, in this study, 63% of our total group demonstrated endothelial dysfunction based on the adult RHI cutoff of 1.67. Similarly, the median RHI = 1.57 in our overall study population is lower than previously described in healthy adolescents (1.78) and children with TIDM (1.63) [32, 46]. In our study, over half of pediatric controls were from a minority racial/ethnic background and a majority from a household with a medium income of less than $50,000 per year. Due to the rarity of disease, families of children with JDM often travel from further distances to seek a specialized academic center and therefore were more likely to represent a racially and financially diverse population in our sample. Put together, these findings raise concern for the risk of endothelial dysfunction in young children, particularly across varying racial and socioeconomic groups. To what extent each of the proposed risk factors (traditional versus sociodemographic versus disease specific) play in the ultimate development of CVD is yet to be determined.

There are several limitations of this work that need to be overcome in further studies. First, the sample size was restricted due to the rarity of JDM, and thus multiple confounding risk factors could not be adjusted for together in a single multivariable analysis, which lacks generalizability of these results. It is also noted that although Endo-PAT is designed to detect the earliest signs of endothelial dysfunction, its prior use in very young children is limited. The feasibility of this test is dependent on cooperation of the child with minimal movement of the extremities during the course of the examination; any movements may have created artifact that could have altered the findings of this study. The Endo-PAT RHI calculation is specifically designed to correct for any detected artifact to assure the validity of the results. To address this concern more diligently, all study reports were examined by the manufacturer for quality assurance.

## Conclusions

With progress in the treatment of rheumatologic diseases rapidly advancing, life expectancy has greatly improved, making complications of premature atherosclerosis a potential cause of morbidity and mortality in these patients. In this study, we have shown that atherogenic risk factors are present in the pediatric population and may be associated with endothelial dysfunction, even at very young ages. However, despite increasing concerns that children with rheumatologic disorders may be at increased risk of developing premature atherosclerosis, traditional and sociodemographic features may play a greater role in the ultimate development of cardiovascular disease. Increased awareness of premature atherosclerotic disease in all young children will allow for increased surveillance of modifiable atherogenic risk factors (both traditional and non-traditional) and the optimization of long-term clinical care.

## Data Availability

The datasets used and/or analyzed during the current study are available from the corresponding author on reasonable request.

## Abbreviations

JDM: Juvenile dermatomyositis
CVD: Cardiovascular disease
SLE: Systemic lupus erythematosus
KD: Kawasaki disease
CIMT: Carotid intima-media thickness
FMD: Flow-mediated dilation
Endo-PAT: Endothelial Pulse Amplitude Testing
T1DM: Type I diabetes mellitus
BMI: Body mass index
TC: Total cholesterol
LDL: Low-density lipoprotein
HDL: High-density lipoprotein
TG: Triglycerides
hsCRP: High-sensitivity C-reactive protein
HOMA-IR: Homeostatic Model Assessment for Insulin Resistance
CPK: Creatine phosphokinase
LDH: Lactate dehydrogenase
AST: Aspartate aminotransferase
vWF: Von-Willebrand factor
CMAS: Childhood Myositis Assessment Scale
DAS: Disease Activity Score
MDI: Myositis Damage Index
RHI: Reactive hyperemia index
CARRA: Childhood Arthritis and Rheumatology Research Alliance
